# The prevalence of educational burnout, depression, anxiety, and stress among medical students of the Islamic Azad University in Tehran, Iran

**DOI:** 10.1186/s12909-021-02874-7

**Published:** 2021-09-05

**Authors:** Ghazal Aghajani Liasi, Sanaz Mahdi Nejad, Nafiseh Sami, Shahrzad Khakpour, Batool Ghorbani Yekta

**Affiliations:** 1 Department of Physiology, Faculty of Medicine, Tehran Medical Sciences, Islamic Azad University of Medical Sciences, Shariati St, Tehran, Iran; 2 Student Research Committee, Tehran Medical Sciences, Islamic Azad University, Tehran, Iran

**Keywords:** Academic exhaustion, Burout, Depression, Anxiety, Stress, Medical student

## Abstract

**Background:**

Psychological disorders have negative consequences on students’ learning and academic performance. In addition, academic burnout is one of the common challenges that affects students’ motivation and academic eagerness; however, the determinant is not clear. Medical students, meanwhile, demand special attention due to their professional responsibilities. In this regard, this study is conducted to investigate the academic burnout, rate of depression, anxiety and stress as well as related factors among undergraduate medical students at the Tehran Medical Sciences Islamic Azad University.

**Methods:**

This cross-sectional and descriptive study was performed on medical students of Islamic Azad University of Tehran in 2017. In phase I, conducted on all stager students, Maslach Burnout questionnaire was used. In phase II, the DASS-42 questionnaire was provided for 123 students, 120 of whom met the inclusion criteria. In addition, another questionnaire including gender, age, lifestyle, marital and financial status, nutrition style, vitamin D deficiency, smoking, study hours per week, work efficiency and distance from the place of residence to the teaching hospital was used. Finally, the data extracted by SPSS version 23 was analyzed at the significance level of 0.05.

**Results:**

In phase I of the study, 17 subjects showed academic burnout (16.3%). Out of all, 76.5% of students with academic burnout did not focus on the study and students’ academic burnout was associated with a decrease in their focus (*P* < 0.05). However, the relationship between academic burnout and other factors was not significant.

In phase II, the prevalence of depression, anxiety and stress was 37.5, 41.1 and 30.3%, respectively. The prevalence of severe and very severe degrees that required psychiatric follow-up were 10.5, 10.5 and 7% for depression, anxiety and stress, respectively. According to statistical analyzes, there is a significant direct relationship between anxiety and the distance from the place of residence to the teaching hospital (*P* = 0.040).

**Conclusion:**

The present study estimated the prevalence of academic burnout to be between 9.2 and 23.4%, considering the 5% error in the calculation, and the level of anxiety was related to the distance from the place of residence to the hospital.

**Supplementary Information:**

The online version contains supplementary material available at 10.1186/s12909-021-02874-7.

## Background

The increasing prevalence of mental health issues has prompted the World Health Organization to dedicate 1 day a year (October 10th) to the world mental health under the slogan: Promotion of Mental Health and Prevention of Suicide [1]. Among mental disorders, 284 million people experienced anxiety disorders, making it the most prevalent mental health disorder with an average of 3.8%. Depression was in second place with a global prevalence of 3.4%, affecting 264 million people worldwide [2].

Given that medical education is one of the most exhausting educational programs, students in this field are more at risk [3]. This educational process requires a specific time and emotional commitment that has negative impacts on mental health when accompanied by stress, leading to depression and anxiety [4, 5].

Medical career burnout is defined as emotional fatigue syndrome and a sense of inefficiency in the medical profession [6]. It can be considered as a precursor to other mental disorders such as depression. Burnout can reduce the quality of life at a higher risk of depression, mostly for physicians [7, 8]. %20 Of medical students have a background of academic depression compared to 8.6% prevalence of burnout in the general population [9, 10]. Recent studies have shown 12% of medical students have suicidal ideas while studying [11, 12]. Additionaly, 1% of men and 2% of women commit suicide [13]. Burnout can also involve occupational manners and ultimately affect patient care. In a study involving medical students, Dyrbye and colleagues showed that students with burnout background are more expected to have unprofessional behavior [14].

Although there is not much research in this field in the Middle East, according to the results of limited studies, the higher rates of mental distress in Eastern compared to Western countries can be related to cultural and religious differences [15]. Several studies have examined stressors for medical students and divided them into three general categories of educational stress and psychosocial as well as health-related issues [16].

Educational stressors include the number of exams, performance on the exams, high self-expectations, too much and difficult educational content, lack of time to review, problems with the study schedule, lack of appropriate study resources, the professional responsibilities, heavy work and study pressure, and lack of time for entertainment [16, 17].

Psychosocial stressors include high parental expectations, loneliness, family problems, being far from home and having to live in dormitories, difficulty in reading reference books, difficulty in traveling back home, financial problems, peer relationships, dormitory living conditions, relationships with the roommates, and lack of personal interest in medicine [16]. As shown in previous studies, there are educational, social and psychological issues concerning students who live in dormitories and away from their family and friends. They may be homesicked, score differently in exams and face some financial difficulties [18].

Health-related stressors include sleep disorders, attending multiple classes, nutrition, exercise, quality of university food, physical illness, smoking, and substance abuse [16]. Moreover, sufficient sleep and regular exercise have increased the life quality of residents and fellowships [19], while poor sleep patterns [20] and low activity [21] are associated with mental conditions such as anxiety and depression.

Exam stress is one of the most significant stressors [16]; however, some degree of stress seems reasonable as a natural part of medical education and even essential for learning [17]. Although stress can be motivating, not all students can manage it [22]. Everyone needs a certain amount of pressure to show the best performance, but when pressure exceeds the ability to cope, stress will result [23].

The effect of mental distress on students’ learning is an important issue. Stress affects learning and academic performance by reducing attention and concentration along with impaired decision-making [3]. On the other hand, anxiety and depression reduce the ability to establish appropriate and reasonable relationships with patients through the reduction of self-confidence [24, 25].

Although the mental health of medical students, as future therapists, is very important, there is not sufficient literature regarding this issue. Therefore, the present study aimed to investigate the academic burnout, evaluate depression, anxiety, and stress among undergraduate medical students of the Tehran Medical Sciences Islamic Azad University in a Middle East setting.

## Methods

### Study design and setting

In this study, the Persian version of both Maslach [26] and DASS questionnaires [27] were used, in addition to a designed demographic questionnaire. The results were analyzed by SPSS 23, considering *P*-value of < 0.05 significant.

### Part I

Maslach questionnaire was distributed among all stager medical students in the Tehran Medical Sciences Islamic Azad University in 2017 to evaluate academic burnout (Undergraduate medical students are called Stagers, when they have 2 years ahead, before internship). The validity and reliability of this questionnaire was confirmed by Rostami et al. on female students of Isfahan University [28].

### Maslach questionnaire

The Maslach questionnaire has a total of 15 items and includes three sub-scales which are academic self-efficacy (six items), emotional fatigue (five items) and skepticism (four items) (see Additional file 1). All statements are graded as seven-point Likert scaling from never (0) to always [6]. The test key for the questions of each scale is as follows:

- Questions 3, 6, 8, 9, 12, and 15 for academic self-efficacy’s sub-scale
- Questions 1, 4, 7, 10, and 13 for emotional fatigue sub-scale
- Questions 2, 5, 11 and 14 for skepticism sub-scale

There are academic inefficiencies which according to utilization of educational efficiency scale (positive sentences), questions of this sub-scale will be numerated in reverse. Based upon, to ensure comparison with previous research, the high rate of burnout was defined as followes:

- Emotional fatigue: score 27 and above
- Skepticism: 10 or higher
- Academic self-efficacy: 33 or less

### Part II

This part was conducted on stager medical students in the Tehran Medical Sciences Islamic Azad University. In this cross-sectional descriptive-analytical study, 123 students completed the DASS questionnaire (depression, anxiety and stress scale), to assess their negative emotional states associated with depression, anxiety, and stress. Another questionnaire on the demographic information such as gender, age, lifestyle, financial status, the distance from the place of residence to the teaching hospital, and the academic average was also prepared according to the secondary objectives of the study (see Additional file 2). The students received the questionnaires and informed consent forms. Students who agreed to participate in the study and completed the questionnaires met the inclusion criteria.

### Depression anxiety stress scales (DASS)

Ali Sahebi confirmed the validity and reliability of the DASS questionnaire for the Iranian population [29]. The DASS questionnaire aims to provide an appropriate and integrated framework for mental health screening. The main form of the questionnaire contains 42 items and examines each of the psychological structures of stress, anxiety, and depression by 14 different questions. The items are answered in a range of four options from ‘never’ to ‘almost always’ using a self-report method. Respondents can choose their answers as one of the options opposite the relevant question. The scoring method is in the form of Likert and from zero to three, in which zero, one, two, and three represent never, sometimes, often, and almost always, respectively (see Additional file 3).

Each item measures one of the scales, and there is no specific sequence for the items. The test key for the questions of each scale is as follows:

- Questions 3, 5, 10, 13, 16, 17, 21, 24, 26, 31, 34, 37, 38, and 42 for depression sub-scale;
- Questions 4, 2, 7, 9, 15, 19, 20, 23, 25, 28, 30, 36, 40, and 41 for anxiety sub-scale; and
- Questions 1, 6, 8, 11, 12, 14, 18, 22, 27, 29, 32, 33, 35, and 39 for stress sub-scale.

The scores related to the items of each scale are summed for interpretation. Total scores are interpreted as follows:

- Depression: 0–9 normal, 10–13 mild, 14–20 moderate, 21–27 severe, and 28^+^ extremely severe;
- Anxiety: 0–7 normal, 8–9 mild, 10–14 moderate, 15–19 severe, and 20^+^ extremely severe; and
- Stress: 0–14 normal, 15–18 mild, 19–25 moderate, 26–33 severe, and 34^+^ extremely severe.

At any scale, severe and extremely severe degrees need psychiatric interventions.

### Statistical analysis

The data of the Maslach questionnaire was analyzed with excel software and SPSS version 23 and the results were presented. The sampling method was counting. Data of the DASS-42 and the attached demographic questionnaire were analyzed by SPSS 23.

First, the sum of scores was calculated and interpreted for each scale based on the specific scoring and grading scale of the DASS-42 questionnaire. The frequency for qualitative variables, mean and standard deviation (SD) for quantitative variables were calculated separately for depression, anxiety, and stress. Finally, the relationship of depression, anxiety, and stress with each of the independent qualitative and quantitative variables was investigated using Chi-square and ANOVA analysis. In addition, logistic regression analysis was also done. *P*-value of < 0.05 was considered significant.

## Results

In this study, all stager medical students of Tehran Medical Sciences Islamic Azad University received Maslach questionnaire and 123 students received the DASS questionnaire.

In the results of part I, 17 of the subjects had academic burnout (16.3%). Considering the 5% error in the calculation, the academic burnout occurence of medical students is between 9.2 to 23.4%. The variables and their associations with educational burnout are presented in Table 1. The sub-scales of the Maslach questionnaire which includes emotional fatigue, academic self-efficacy and skepticism were estimated to be 17.8, 98.2 and 4.5%, respectively. 80.2% of people with burnout were women. According to the statistical test, the relationship between gender and academic burnout was not significant.

**Table 1:** Important variables and their associations with academic burnout

Variables		Without burnout N(%)	With burnout N(%)	*P* .value
Gender	Female	69 (80.2%)	15 (88.2%)	0.437
	Male	17 (19.8%)	2 (11.8%)	
Marital status	Single	72 (82.8%)	14 (82.4%)	0.894
	Married	15 (17.2%)	3 (17.6%)	
Family income	< 20 million Rials	3 (3.5%)	2 (11.8%)	> 0.05
	20–50 million Rials	31 (36.5%)	6 (35.3%)	
	> 50 million Rials	51 (60.0%)	9 (52.9%)	

From the total burnout, 82.4% were single and 17.6% were married. Chi-square test showed that there is no significant relationship between academic burnout and marital status. In addition, Income of less than 20 million Rial, 20 to 50 million Rial and above 50 million Rial were reported by 11.8, 35.3 and 52.9%, respectively. Among other students, this figure was estimated to be 3.5, 36.5 and 60%, respectively. However, Chi-square test showed that no significant relationship was found between family income status and academic burnout. Moreover, according to the statistical test, the relationship between academic block and academic burnout was not significant.

Out of the total burnout, 76.5% showed decreased concentration during the study. This figure was estimated at 42.5% in other students. Thus, students’ academic burnout is associated with a decrease in their concentration (*p* < 0.05).

Less than 5 h, 5 to 7 h and more than 7 h of sleep were stated in 11.8, 70.6 and 17.6% of students with academic burnout, respectively. Among other students, these figures were 16.5, 55.3 and 28.2%, respectively.

Poor appetite was indicated in 0.0 and 5.9% of people with and without burnout, respectively.

Chi-square test showed that there is no significant relationship between course renewal, student’s leisure time and sleep, amount of study hours per week, daily fatigue, diet, smoking cigarette, and academic burnout. In addition, no significant relationship was found between appetite, vitamin D deficiency, efficiency in assignments and burnout.

In part II, the response rate was 97.5% and 120 students met inclusion criteria consisting of written consent and completion of the questionnaire. Of this population, 96 (80.7%) were female, and 23 (19.3%) were male. The mean age of the sample was 24.4 + 2.06 years, and students were in the age range of 20 to 34 years.

Interpretation of the DASS questionnaire data indicated that of the study participants, 36 students (37.5%) suffered depression. Depression was extremely severe in 4 (4.2%), severe in 6 (6.3%), moderate in 13 (13.5%), and mild in 13 students (13.5%). Normal conditions were reported in 60 (62.5%) students. Of the study participants, 39 students (41.1%) suffered anxiety. Anxiety was extremely severe in 2 (2.1%), severe in 8 (8.4%), moderate in 15 (15.8%), and mild in 14 students (14.7%). Normal conditions were reported in 56 (58.9%) students. Of the study participants, 30 students (30.3%) suffered stress. Stress was extremely severe in 1 (0.1%), severe in 6 (0.6%), moderate in 11 (11.1%), and mild in 12 students (12.1%). Normal conditions were reported in 69 (69.7%) students. The overall prevalence of mental disorders was 36.3% in the study population. The prevalence of severe and extremely severe degrees that require psychiatric intervention according to the questionnaire was 10.5% (*n* = 10), 10.5% (*n* = 10), and 7.1% (*n* = 7) for depression, anxiety, and stress, respectively. The mean scores of depression, anxiety, and stress were 9.3 + 8.08, 6.96 + 5.4, and 11.37 + 7.8, respectively as they are reported in Table 2. Statistical analyses confirmed the correlation between depression, anxiety, and stress.

**Table 2:** Descriptive results of depression, anxiety and stress

	Mean	Standard Deviation	Percentage				
			Normal	Mild	Moderate	Severe	Extremely severe
Depression	9.13	8.06	62.5	13.5	13.5	6.3	4.2
Anxiety	6.96	5.40	58.9	14.7	15.8	8.4	2.1
Stress	11.37	7.80	69.7	12.1	11.1	6.1	1.0

The distance from the place of residence to the teaching hospital was short for 7.5% (< 10 km), average for 40.0% (10–20 km), and long for 52.5% (> 20 km). According to statistical analyzes, there was a significant difference between the distance from the place of residence to the hospital and anxiety (*P* = 0.040). However, it was not correlated with depression and stress. According to statistical data on the students’ lifestyle, 78.0, 4.2, and 17.8% lived with their parents, in a dormitory, and in an independent home, respectively. According to the results, there was no significant relationship between lifestyle and depression, anxiety, and stress.

According to the statistical data in terms of financial status, 5.1% earned less than 20 million Rials, 36.8% between 20 to 50 million Rials, and 58.1% more than 50 million Rials per month. No significant relationship was found between financial status and depression, anxiety, and stress. As mentioned, 80.7% of the second part participants were female, and 19.3% were male. Chi-square statistical analyses revealed no significant relationship between gender and depression, anxiety, and stress. The mean age of the sample was 24.4 + 2.06 years, and students were in the age range of 20 to 34 years. The highest frequency was related to the 25-year-old age group, which accounted for 28.2% of the study population. According to ANOVA analysis, there was no significant relationship between age and depression, anxiety, and stress.

The students’ academic average was 16 + 0.95. According to ANOVA analysis, no significant relationship was found between students’ academic average and depression, anxiety, and stress. All of the demographic variables are reported in Table 3.

**Table 3:** Demographic variables and their association with depression, anxiety and stress

Variables		Depression		Anxiety		Stress	
		%	*P* .value	%	*P* .value	%	*P* .value
Gender	Female	78.9	0.71	80.9	0.59	79.6	0.15
	Male	21.1		19.1		20.4	
Distance from the place of residence to the teaching hospital	Short (< 10 km)	9.4	0.05	6.3	0.04	8.1	0.11
	Average (10–20 km)	43.8		45.3		42.4	
	Long (> 20 km)	46.9		48.4		49.5	
Lifestyle	With their parents	75.5	0.19	77.7	0.70	76.5	0.61
	In a dormitory	5.3		5.3		5.1	
	In an independent house	19.1		17		18.4	
Financial status	< 20 million Rials	6.4	0.56	6.4	0.99	5.2	0.53
	20–50 million Rials	38.3		39.4		38.1	
	> 50 million Rials	55.3		54.3		56.7	
		Mean ± SD		Mean ± SD		Mean ± SD	
Age		24.4 ± 0.99	0.97	24 ± 1.3	0.45	23.66 ± 0.81	0.31
Academic average		16.38 ± 1.08	0.46	16.47 ± 0.78	0.16	16.73 ± 0.71	0.15

*Abbreviations*: *km* Kilometer, *SD* Standard Deviation

Logistic regression analysis revealed that none of the variables, including gender, marital status, income, distance from the place of residence to the teaching hospital, lifestyle, etc., are predictors of students’ depression, anxiety, stress or academic burnout.

## Discussion

This study was conducted on stager medical students in the Tehran Medical Sciences Islamic Azad University. In our study, student’s academic burnout was related to reduced concentration. In contrast, academic burnout was not found to be significantly associated with gender, study hours per week, marital status, efficiency in assignments, income status, vitamin D deficiency, nutrition style, alarm status and smoking of students. This outcome can be due to sample size and limitaions which will be further discussed. Moreover, studies concerning more than one university and even including different cities can be more precise in this subject.

Among the studied factors, the more distance from the place of residence to the hospital, the more anxiety students had. This relationship is justifiable considering the location of the Tehran Medical Sciences Branch of the Islamic Azad University and its affiliated hospitals in the crowded and densely populated parts of the city, which made transportation difficult. On the other hand, unlike many other fields of study, medical education needs regular and continuous attendance at the university and the hospital, which results in a lot of commuting on most days of the week. This difficult and frequent transportation, which requires a lot of time, money, and energy, can illustrate the significant relationship between the distance from the place of residence to the hospital and anxiety.

Muzafar et al. examined academic depression and its impact among 777 Pakistani medical students. Students were asked to complete the Copenhagen Career Questionnaire. According to the findings, 30.6% of students indicted high academic depression, while our results showed 37.5% deppression. Differences in measurement utility, individual, sample size, social and climatic differences can be the reasons for the discrepancies between studies [30]. Sedighi et al. studied the mental health status of medical students of Rafsanjan University of Medical Sciences and found out that financial problems, changes in sleep habits, and duration of study affected mental health status significantly and negatively [31]. The differences between studies can be due to regional factors, while considering the sample size diversity.

Moutinho Ivana et al. examined the prevalence of depression, anxiety, stress, and related factors in medical students in Brazil. Out of 1009 students, 761 voluntarily completed the questionnaire. The results of this study were in line with the present study in terms of the overall prevalence and the prevalence of severe and extremely severe degrees [32]. Their findings suggested that gender (female) was directly related to depression and stress. This result was not confirmed in the present paper, which can be due to differences in socio-cultural factors, sample size, and gender frequency distribution.

In general, many studies have examined the relationship between gender and mental distress. A systematic review study found that half of the studies examining this association among medical students confirmed the effect of gender on mental disorders, and the other half rejected it. Therefore, this relationship remains controversial [33]. Interestingly, this study examined the correlations between depression, anxiety, and stress and found a high correlation [32], which was also confirmed in the present study.

Paula et al. reviewed the prevalence of anxiety, depression, and suicidal ideation among students systematically. Their study included 48 articles from 40 different countries from 2013 to 2018, resulting in a total population of 56,816 students. The results showed that the prevalence of all three categories of symptoms (anxiety, depression, and suicidal ideation) was higher among students in health-related fields compared to other students [34]. Second, mental disorders are considered a stigma and a sign of weakness both for the patients and their families. This is, in turn, a barrier to psychological interventions and thus increases the prevalence of mental disorders [35].

In the present study, a wide range of variables were considered. However, there were some limitations; This study cannot evaluate the cause-and-effect relationships, the questionnaires were filled voluntarily and by the students themselves; So, this may cause for psychologically disturbed students not to participate, or they may not answer truthfully.

Future researchers can conduct longitudinal, prospective, or retrospective studies to examine the cause-and-effect relationships in more depth. Besides, a comparison can be made between public (governmental) universities that offer free education and private and non-profit universities, which require relatively high tuition, particularly in the field of medicine. Such comparisons can be useful in examining the relationship between tuition and economic status with mental distress. It is also useful to consider medical students in other years of medicine such as medical basic sciences and physiopathology (first 5 years of medical school in Iran), and also internship to get deeper insights into the effect of the curriculum on mental distress. In this regard, factors such as educational pressure (clinical and non-clinical courses), evaluation methods (theoretical or practical), frequency of exams (monthly, quarterly, semester, and annual), and their relationship with students’ academic performance can be investigated. It is possible to go further and examine the extent of medical errors, forensic complaints, and over-diagnostic as well as over-treatment medical measures, which impose high costs on the national health system, according to different degrees of mental distress.

## Conclusion

The prevalence of burnout in this study was estimated to be between 9.2 and 23.4%. The sub-scales of the Maslach questionnaire including emotional fatigue, academic self-efficacy and skepticism were evaluated. Academic burnout was also related to reduced concentration. In addition, the results confirmed the high prevalence of depression, anxiety, and stress among medical students, while anxiety showed the highest prevalence. A significant and direct relationship between anxiety and the distance from the place of residence to the hospital was found. Through further investigation regarding this area, it is possible to determine the disposing, causing and exacerbating factors conserning mental health; therefore, make the necessary changes in the curriculum, along with the provision of better support counseling services. Eventually, efforts should be made to prevent and control psychological conditions to imptove the quality of medical education. Consequently, it is possible to train physically healthier physicians with more scientific and practical knowledge to serve the community.

## Supplementary Information

**Additional file 1.** Maslach Burnout Inventory–Student Survey

**Additional file 2.** Demographic questionnaire.

**Additional file 3.** Depression Anxiety Stress Scales (DASS) questionnaire.

## Data Availability

Data sharing is not applicable to this article as no datasets were generated or analysed during the current study.

## Abbreviation

DASS: Depression, anxiety and stress scale
