# Supplementary material for: The prevalence of educational burnout, depression, anxiety, and stress among medical students of the Islamic Azad University in Tehran, Iran
Source: BMC Med Educ. 2021 Sep 5;21:471. doi: 10.1186/s12909-021-02874-7 (PMC8418739; doi:10.1186/s12909-021-02874-7)
Supplement: Supplementary file 1 — Additional file 1. Maslach Burnout Inventory–Student Survey [file 12909_2021_2874_MOESM1_ESM.docx]

**The Prevalence of Educational Burnout, Depression, Anxiety, and Stress among medical students of the Islamic Azad University in Tehran, Iran**

Qazal Aghajani Elyasi^a^, Sanaz Mahdi Nejad^a^, Nafiseh Sami^b^, Shahrzad Khakpour^a^, Batool Ghorbani Yekta^a*^

^a^Department of Physiology, Faculty of Medicine, Tehran Medical Sciences, Islamic Azad University, Tehran, Iran

^b^Student Research Committee, Faculty of Medicine, Tehran Medical Sciences, Islamic Azad University, Tehran, Iran.

^*^**Address correspondence to:** Batool Ghorbani Yekta, Islamic Azad University of Medical Sciences, Shariati St, Tehran, Iran. Phone: +982122006660, Fax: +982122600712. E-mail: yekta@iautmu.ac.ir

**Maslach Burnout Inventory–Student Survey**

Please give a number from 0 to 6 to each statement, according to the following table.

| 0 | 1 | 2 | 3 | 4 | 5 | 6 |
| --- | --- | --- | --- | --- | --- | --- |
| Never | Seldom  (Few times a year or less) | Now and Then  (Few times a month or less) | Regular  (Few times a month) | Often  (Once a week) | Very often  (few times a week) | Always |

Emotional Fatigue

1. I feel emotionally drained by my studies.

4. I feel used up at the end of a day at school.

7. I feel burned out from my studies.

10. I feel tired when I get up in the morning and I have to face another day at school.

13. Studying or attending a class is really a strain for me.

Skepticism

2. I have become less interested in my studies since my enrollment at the school.

5. I have become less enthusiastic about my studies.

11. I have become more cynical about the potential usefulness of my studies.

14. I doubt the significance of my studies.

Academic Efficacy

3. I can effectively solve the problems that arise in my studies.

6. I believe that I make an effective contribution to the classes that I attend.

8. In my opinion, I am a good student.

9. I have learned many interesting things during the course of my studies.

12. I feel stimulated when I achieve my study goals.

15. During class I feel confident that I am effective in getting things done.
