# Supplementary material for: The prevalence of educational burnout, depression, anxiety, and stress among medical students of the Islamic Azad University in Tehran, Iran
Source: BMC Med Educ. 2021 Sep 5;21:471. doi: 10.1186/s12909-021-02874-7 (PMC8418739; doi:10.1186/s12909-021-02874-7)
Supplement: Supplementary file 2 — Additional file 2. Demographic questionnaire. [file 12909_2021_2874_MOESM2_ESM.docx]

**The Prevalence of Educational Burnout, Depression, Anxiety, and Stress among medical students of the Islamic Azad University in Tehran, Iran**

Qazal Aghajani Elyasi^a^, Sanaz Mahdi Nejad^a^, Nafiseh Sami^b^, Shahrzad Khakpour^a^, Batool Ghorbani Yekta^a*^

^a^Department of Physiology, Faculty of Medicine, Tehran Medical Sciences, Islamic Azad University, Tehran, Iran

^b^Student Research Committee, Faculty of Medicine, Tehran Medical Sciences, Islamic Azad University, Tehran, Iran.

^*^**Address correspondence to:** Batool Ghorbani Yekta, Islamic Azad University of Medical Sciences, Shariati St, Tehran, Iran. Phone: +982122006660, Fax: +982122600712. E-mail: yekta@iautmu.ac.ir

1- Age (Please write in numbers)

2- Gender

3- Marital status

4- Distance from the place of residence to the teaching hospital:

- Less than 10 kilometers
- Between 10-20 kilometers
- More than 20 kilometers

5- I live … .

- With my parents
- In a dormitory
- In an independent house

6- My average family income is …. per month.

- Less than 20 million Rials
- Between 20-50 million Rials
- More than 50 million Rials

7- My academic average is … . (Please write in numbers)

8- My academic block is … . (Please write in numbers)

9- I can concentrate easily when I study.

- Yes
- No

10- I usually sleep … a day.

- 5 hours
- Between 5-7 hours
- More than 7 hours

11- I usually study … per week.

- I just study the night before the exam
- 15 hours
- 30 hours
- 40 hours

12- I have efficiency in my assignments,

- Yes
- No

13- I think my appetite is … .

- Poor
- Average
- Good

14- I have a lot of … in my usual diet.

- Fast-food
- Low-calorie foods
- High-calorie foods
- No specific style

15- I have experienced course renewal.

- Yes
- No

16- I smoke cigarette.

- Yes
- No

17- I usually feel tired during the day.

- Yes
- No

18- I have experienced vitamin D deficiency.

- Yes
- No

19- I usually have … of leisure time per day.

- Less than 3 hours
- Between 3-5 hours
- More than 5 hours
