# Supplementary material for: The prevalence of educational burnout, depression, anxiety, and stress among medical students of the Islamic Azad University in Tehran, Iran
Source: BMC Med Educ. 2021 Sep 5;21:471. doi: 10.1186/s12909-021-02874-7 (PMC8418739; doi:10.1186/s12909-021-02874-7)
Supplement: Supplementary file 3 — Additional file 3. Depression Anxiety Stress Scales (DASS) questionnaire. [file 12909_2021_2874_MOESM3_ESM.docx]

**The Prevalence of Educational Burnout, Depression, Anxiety, and Stress among medical students of the Islamic Azad University in Tehran, Iran**

Qazal Aghajani Elyasi^a^, Sanaz Mahdi Nejad^a^, Nafiseh Sami^b^, Shahrzad Khakpour^a^, Batool Ghorbani Yekta^a*^

^a^Department of Physiology, Faculty of Medicine, Tehran Medical Sciences, Islamic Azad University, Tehran, Iran

^b^Student Research Committee, Faculty of Medicine, Tehran Medical Sciences, Islamic Azad University, Tehran, Iran.

^*^**Address correspondence to:** Batool Ghorbani Yekta, Islamic Azad University of Medical Sciences, Shariati St, Tehran, Iran. Phone: +982122006660, Fax: +982122600712. E-mail: yekta@iautmu.ac.ir

Over the past week …

1. I found myself getting upset by quite trivial things

- Did not apply to me at all - NEVER
- Applied to me to some degree, or some of the time - SOMETIMES
- Applied to me to a considerable degree, or a good part of time - OFTEN
- Applied to me very much, or most of the time - ALMOST ALWAYS

2. I was aware of dryness of my mouth

- Did not apply to me at all - NEVER
- Applied to me to some degree, or some of the time - SOMETIMES
- Applied to me to a considerable degree, or a good part of time - OFTEN
- Applied to me very much, or most of the time - ALMOST ALWAYS

3. I couldn’t seem to experience any positive feeling at all

- Did not apply to me at all - NEVER
- Applied to me to some degree, or some of the time - SOMETIMES
- Applied to me to a considerable degree, or a good part of time - OFTEN
- Applied to me very much, or most of the time - ALMOST ALWAYS

4. I experienced breathing difficulty (eg, excessively rapid breathing, breathlessness in the absence of physical exertion)

- Did not apply to me at all - NEVER
- Applied to me to some degree, or some of the time - SOMETIMES
- Applied to me to a considerable degree, or a good part of time - OFTEN
- Applied to me very much, or most of the time - ALMOST ALWAYS

5. I just couldn't seem to get going

- Did not apply to me at all - NEVER
- Applied to me to some degree, or some of the time - SOMETIMES
- Applied to me to a considerable degree, or a good part of time - OFTEN
- Applied to me very much, or most of the time - ALMOST ALWAYS

6. I tended to over-react to situations

- Did not apply to me at all - NEVER
- Applied to me to some degree, or some of the time - SOMETIMES
- Applied to me to a considerable degree, or a good part of time - OFTEN
- Applied to me very much, or most of the time - ALMOST ALWAYS

7. I had a feeling of shakiness (eg, legs going to give way)

- Did not apply to me at all - NEVER
- Applied to me to some degree, or some of the time - SOMETIMES
- Applied to me to a considerable degree, or a good part of time - OFTEN
- Applied to me very much, or most of the time - ALMOST ALWAYS

8. I found it difficult to relax

- Did not apply to me at all - NEVER
- Applied to me to some degree, or some of the time - SOMETIMES
- Applied to me to a considerable degree, or a good part of time - OFTEN
- Applied to me very much, or most of the time - ALMOST ALWAYS

9. I found myself in situations that made me so anxious I was most relieved when they ended

- Did not apply to me at all - NEVER
- Applied to me to some degree, or some of the time - SOMETIMES
- Applied to me to a considerable degree, or a good part of time - OFTEN
- Applied to me very much, or most of the time - ALMOST ALWAYS

10. I felt that I had nothing to look forward to

- Did not apply to me at all - NEVER
- Applied to me to some degree, or some of the time - SOMETIMES
- Applied to me to a considerable degree, or a good part of time - OFTEN
- Applied to me very much, or most of the time - ALMOST ALWAYS

11. I found myself getting upset rather easily

- Did not apply to me at all - NEVER
- Applied to me to some degree, or some of the time - SOMETIMES
- Applied to me to a considerable degree, or a good part of time - OFTEN
- Applied to me very much, or most of the time - ALMOST ALWAYS

12. I felt that I was using a lot of nervous energy

- Did not apply to me at all - NEVER
- Applied to me to some degree, or some of the time - SOMETIMES
- Applied to me to a considerable degree, or a good part of time - OFTEN
- Applied to me very much, or most of the time - ALMOST ALWAYS

13. I felt sad and depressed

- Did not apply to me at all - NEVER
- Applied to me to some degree, or some of the time - SOMETIMES
- Applied to me to a considerable degree, or a good part of time - OFTEN
- Applied to me very much, or most of the time - ALMOST ALWAYS

14. I found myself getting impatient when I was delayed in any way (eg, lifts, traffic lights, being kept waiting)

- Did not apply to me at all - NEVER
- Applied to me to some degree, or some of the time - SOMETIMES
- Applied to me to a considerable degree, or a good part of time - OFTEN
- Applied to me very much, or most of the time - ALMOST ALWAYS

15. I had a feeling of faintness

- Did not apply to me at all - NEVER
- Applied to me to some degree, or some of the time - SOMETIMES
- Applied to me to a considerable degree, or a good part of time - OFTEN
- Applied to me very much, or most of the time - ALMOST ALWAYS

16. I felt that I had lost interest in just about everything

- Did not apply to me at all - NEVER
- Applied to me to some degree, or some of the time - SOMETIMES
- Applied to me to a considerable degree, or a good part of time - OFTEN
- Applied to me very much, or most of the time - ALMOST ALWAYS

17. I felt I wasn't worth much as a person

- Did not apply to me at all - NEVER
- Applied to me to some degree, or some of the time - SOMETIMES
- Applied to me to a considerable degree, or a good part of time - OFTEN
- Applied to me very much, or most of the time - ALMOST ALWAYS

18. I felt that I was rather touchy

- Did not apply to me at all - NEVER
- Applied to me to some degree, or some of the time - SOMETIMES
- Applied to me to a considerable degree, or a good part of time - OFTEN
- Applied to me very much, or most of the time - ALMOST ALWAYS

19. I perspired noticeably (eg, hands sweaty) in the absence of high temperatures or physical exertion

- Did not apply to me at all - NEVER
- Applied to me to some degree, or some of the time - SOMETIMES
- Applied to me to a considerable degree, or a good part of time - OFTEN
- Applied to me very much, or most of the time - ALMOST ALWAYS

20. I felt scared without any good reason

- Did not apply to me at all - NEVER
- Applied to me to some degree, or some of the time - SOMETIMES
- Applied to me to a considerable degree, or a good part of time - OFTEN
- Applied to me very much, or most of the time - ALMOST ALWAYS

21. I felt that life wasn't worthwhile

- Did not apply to me at all - NEVER
- Applied to me to some degree, or some of the time - SOMETIMES
- Applied to me to a considerable degree, or a good part of time - OFTEN
- Applied to me very much, or most of the time - ALMOST ALWAYS

22. I found it hard to wind down

- Did not apply to me at all - NEVER
- Applied to me to some degree, or some of the time - SOMETIMES
- Applied to me to a considerable degree, or a good part of time - OFTEN
- Applied to me very much, or most of the time - ALMOST ALWAYS

23. I had difficulty in swallowing

- Did not apply to me at all - NEVER
- Applied to me to some degree, or some of the time - SOMETIMES
- Applied to me to a considerable degree, or a good part of time - OFTEN
- Applied to me very much, or most of the time - ALMOST ALWAYS

24. I couldn't seem to get any enjoyment out of the things I did

- Did not apply to me at all - NEVER
- Applied to me to some degree, or some of the time - SOMETIMES
- Applied to me to a considerable degree, or a good part of time - OFTEN
- Applied to me very much, or most of the time - ALMOST ALWAYS

25. I was aware of the action of my heart in the absence of physical exertion (eg, sense of heart rate increase, heart missing a beat)

- Did not apply to me at all - NEVER
- Applied to me to some degree, or some of the time - SOMETIMES
- Applied to me to a considerable degree, or a good part of time - OFTEN
- Applied to me very much, or most of the time - ALMOST ALWAYS

26. I felt down-hearted and blue

- Did not apply to me at all - NEVER
- Applied to me to some degree, or some of the time - SOMETIMES
- Applied to me to a considerable degree, or a good part of time - OFTEN
- Applied to me very much, or most of the time - ALMOST ALWAYS

27. I found that I was very irritable

- Did not apply to me at all - NEVER
- Applied to me to some degree, or some of the time - SOMETIMES
- Applied to me to a considerable degree, or a good part of time - OFTEN
- Applied to me very much, or most of the time - ALMOST ALWAYS

28. I felt I was close to panic

- Did not apply to me at all - NEVER
- Applied to me to some degree, or some of the time - SOMETIMES
- Applied to me to a considerable degree, or a good part of time - OFTEN
- Applied to me very much, or most of the time - ALMOST ALWAYS

29. I found it hard to calm down after something upset me

- Did not apply to me at all - NEVER
- Applied to me to some degree, or some of the time - SOMETIMES
- Applied to me to a considerable degree, or a good part of time - OFTEN
- Applied to me very much, or most of the time - ALMOST ALWAYS

30. I feared that I would be 'thrown' by some trivial but unfamiliar task

- Did not apply to me at all - NEVER
- Applied to me to some degree, or some of the time - SOMETIMES
- Applied to me to a considerable degree, or a good part of time - OFTEN
- Applied to me very much, or most of the time - ALMOST ALWAYS

31. I was unable to become enthusiastic about anything

- Did not apply to me at all - NEVER
- Applied to me to some degree, or some of the time - SOMETIMES
- Applied to me to a considerable degree, or a good part of time - OFTEN
- Applied to me very much, or most of the time - ALMOST ALWAYS

32. I found it difficult to tolerate interruptions to what I was doing

- Did not apply to me at all - NEVER
- Applied to me to some degree, or some of the time - SOMETIMES
- Applied to me to a considerable degree, or a good part of time - OFTEN
- Applied to me very much, or most of the time - ALMOST ALWAYS

33. I was in a state of nervous tension

- Did not apply to me at all - NEVER
- Applied to me to some degree, or some of the time - SOMETIMES
- Applied to me to a considerable degree, or a good part of time - OFTEN
- Applied to me very much, or most of the time - ALMOST ALWAYS

34. I felt I was pretty worthless

- Did not apply to me at all - NEVER
- Applied to me to some degree, or some of the time - SOMETIMES
- Applied to me to a considerable degree, or a good part of time - OFTEN
- Applied to me very much, or most of the time - ALMOST ALWAYS

35. I was intolerant of anything that kept me from getting on with what I was doing

- Did not apply to me at all - NEVER
- Applied to me to some degree, or some of the time - SOMETIMES
- Applied to me to a considerable degree, or a good part of time - OFTEN
- Applied to me very much, or most of the time - ALMOST ALWAYS

36. I felt terrified

- Did not apply to me at all - NEVER
- Applied to me to some degree, or some of the time - SOMETIMES
- Applied to me to a considerable degree, or a good part of time - OFTEN
- Applied to me very much, or most of the time - ALMOST ALWAYS

37. I could see nothing in the future to be hopeful about

- Did not apply to me at all - NEVER
- Applied to me to some degree, or some of the time - SOMETIMES
- Applied to me to a considerable degree, or a good part of time - OFTEN
- Applied to me very much, or most of the time - ALMOST ALWAYS

38. I felt that life was meaningless

- Did not apply to me at all - NEVER
- Applied to me to some degree, or some of the time - SOMETIMES
- Applied to me to a considerable degree, or a good part of time - OFTEN
- Applied to me very much, or most of the time - ALMOST ALWAYS

39. I found myself getting agitated

- Did not apply to me at all - NEVER
- Applied to me to some degree, or some of the time - SOMETIMES
- Applied to me to a considerable degree, or a good part of time - OFTEN
- Applied to me very much, or most of the time - ALMOST ALWAYS

40. I was worried about situations in which I might panic and make a fool of myself

- Did not apply to me at all - NEVER
- Applied to me to some degree, or some of the time - SOMETIMES
- Applied to me to a considerable degree, or a good part of time - OFTEN
- Applied to me very much, or most of the time - ALMOST ALWAYS

41. I experienced trembling (eg, in the hands)

- Did not apply to me at all - NEVER
- Applied to me to some degree, or some of the time - SOMETIMES
- Applied to me to a considerable degree, or a good part of time - OFTEN
- Applied to me very much, or most of the time - ALMOST ALWAYS

42. I found it difficult to work up the initiative to do things

- Did not apply to me at all - NEVER
- Applied to me to some degree, or some of the time - SOMETIMES
- Applied to me to a considerable degree, or a good part of time - OFTEN
- Applied to me very much, or most of the time - ALMOST ALWAYS
